# Dichloridobis{1-[(2-methyl­benzimidazol-1-yl-κ*N* ^3^)meth­yl]benzotriazole}zinc

**DOI:** 10.1107/S160053681203526X

**Published:** 2012-08-15

**Authors:** Lianqing Song, Meihong Zhao, Guoshi Cui, Jianghua Gao, Lin Lin

**Affiliations:** a Department of Chemistry, Henan Institute of Education, Zhengzhou 450046, People’s Republic of China; b Isotope Institute of Henan Acacemy of Sciences, Zhengzhou 450052, People’s Republic of China; c Henan Engineering Research Center For Electron Beam Application, Zhengzhou 450052, People’s Republic of China; d Zhengzhou Huitong Advertisement Material Co., Ltd, Zhengzhou 50064, People’s Republic of China; e Light Industry Mechanical Institute of Henan Province, Luoyang 471099, People’s Republic of China

## Abstract

The title mononuclear Zn^II^ complex, [ZnCl_2_(C_15_H_13_N_5_)_2_], is isotypic with the previously reported Hg^II^ complex. The Zn^II^ atom is located on a twofold rotation axis and has a distorted tetra­hedral environment of two Cl atoms and two N atoms from two heterocyclic ligands. In the crystal, complex mol­ecules are extended by inter­molecular π–π inter­actions [centroid–centroid distance = 3.792 (2) Å] into a three-dimensional supra­molecular network.

## Related literature

For background information on complexes constructed from *N*-heterocyclic ligands, see: Liu *et al.* (2012 ▶); Bondar *et al.* (2012 ▶); Shao *et al.* (2008 ▶); Su *et al.* (2003 ▶). For the isotypic Hg^II^ complex, see: Wu *et al.* (2009 ▶).
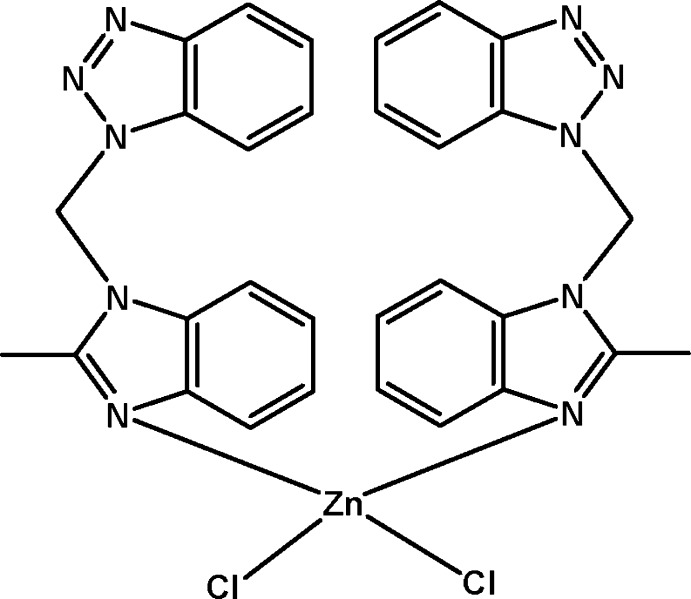


## Experimental

### 

#### Crystal data

[ZnCl_2_(C_15_H_13_N_5_)_2_]
*M* *_r_* = 662.90
Monoclinic, 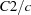

*a* = 15.721 (4) Å
*b* = 12.617 (4) Å
*c* = 14.728 (3) Å
β = 99.13 (3)°
*V* = 2884.3 (13) Å^3^
*Z* = 4
Mo *K*α radiation
μ = 1.08 mm^−1^
*T* = 295 K
0.22 × 0.20 × 0.20 mm

#### Data collection

Rigaku Saturn CCD diffractometer
Absorption correction: multi-scan (*CrystalClear*; Rigaku/MSC, 2006 ▶) *T*_min_ = 0.797, *T*_max_ = 0.813
17560 measured reflections
3423 independent reflections
2977 reflections with *I* > 2σ(*I*)
*R*_int_ = 0.041

#### Refinement

*R*[*F* ^2^ > 2σ(*F* ^2^)] = 0.050
*wR*(*F* ^2^) = 0.137
*S* = 1.07
3423 reflections
196 parameters
H-atom parameters constrained
Δρ_max_ = 0.25 e Å^−3^
Δρ_min_ = −0.30 e Å^−3^

### 

Data collection: *CrystalClear* (Rigaku/MSC, 2006 ▶); cell refinement: *CrystalClear*; data reduction: *CrystalClear*; program(s) used to solve structure: *SHELXS97* (Sheldrick, 2008 ▶); program(s) used to refine structure: *SHELXS97* (Sheldrick, 2008 ▶); molecular graphics: *SHELXTL* (Sheldrick, 2008 ▶); software used to prepare material for publication: *SHELXTL*.

## Supplementary Material

Crystal structure: contains datablock(s) global, I. DOI: 10.1107/S160053681203526X/rk2368sup1.cif

Structure factors: contains datablock(s) I. DOI: 10.1107/S160053681203526X/rk2368Isup2.hkl

Additional supplementary materials:  crystallographic information; 3D view; checkCIF report

## Figures and Tables

**Fig. 1.**
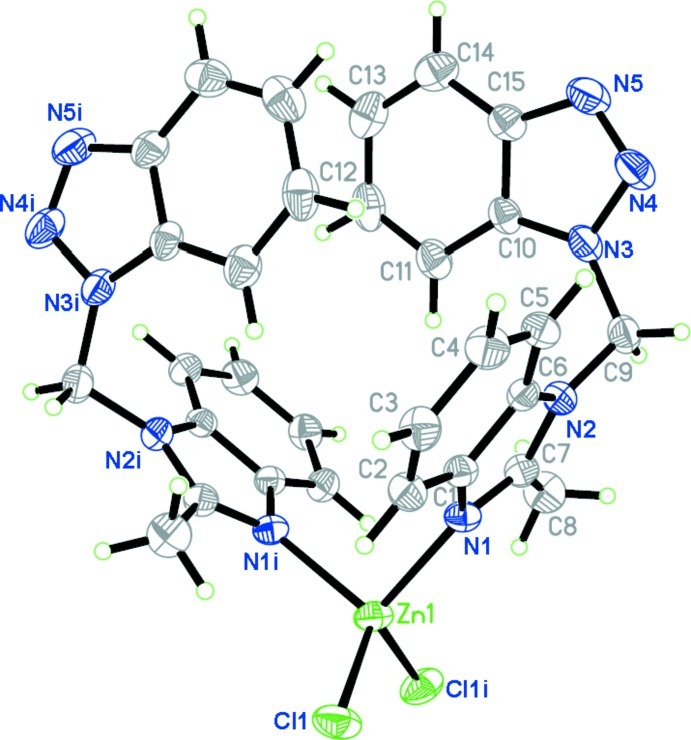
Molecular structure of the title complex with the atom numbering scheme. Displacement ellipsolids are drawn at 30% probability level. Symmetry code: (i) -x, y, 1/2-z.

**Fig. 2.**
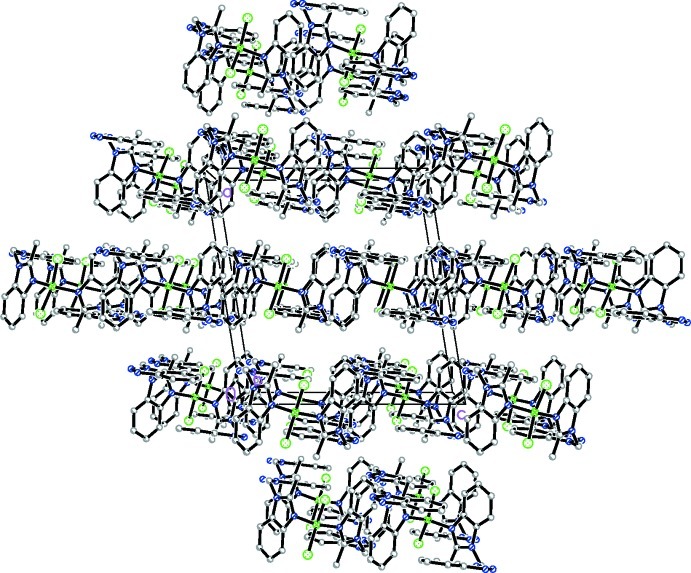
View of the crystal packing of the title complex, showing the three-dimensional supramolecular structure.

## supplementary crystallographic information

### Comment

Multidentate organic ligands have received extensive attention in supramolecular chemistry due to their abilities in constructing novel architectures with interesting properties (Liu *et al.*, 2012; Bondar *et al.*, 2012). Among them, the ligands bearing benzotriazole or benzimidazole groups are good candidates because of their various coordiantion modes and biological activities (Shao *et al.*, 2008; Su *et al.*, 2003). The 1-(2-methylbenzoimidazol-3-yl-methyl)-benzotriazole, simultaneously has the benzotriazole group and the benzoimidazole group, which can be an excellent ligand to form new structures. In this work, we selected this ligand as linker to self-assemble with ZnCl_2_ and obtained the title mononuclear complex, ZnCl_2_(C_15_H_13_N_5_)_2_, which is isostructural with the previously reported Hg^II^ complex (Wu *et al.*, 2009). The Zn atom placed on twofold axis. As shown in Fig. 1, the Zn^II^ is in a distorted tetrahedral geometry and coordinated by two Cl atoms and two N atoms from two 1-(2-methylbenzimidazol-1-yl-methyl)benzotriazole ligands. Because the 2-position substituent methyl of benzimidazole ring is an electrondonating group, the N atom of benzimidazole ring has higher electron density than others. Therefore, the N atom of benzimidazole ring is prior to coordinate with metal ions, which leads to the ligand adopting a monodentate fashion. In addition, intramolecular π–π interactions between the imidazole rings and phenyl rings of benzimidazole rings (centroid-to-centroid separation: 3.631 (19)Å), intermolecular π–π interactions between phenyl rings of benzotriazole rings (centroid-to-centroid separation: 3.792 (2)Å) consolidate the crystal packing, as depicted in Fig. 2.

### Experimental

Synthesis of ZnCl_2_(C_15_H_13_N_5_)_2_: a methanol solution (4 ml) of ligand 1-(2-methyl-benzoimidazol-3-yl-methyl)-benzotriazole (26.3 mg, 0.1 mmol) was added dropwise to the methanol solution (5 ml) of ZnCl_2_ (13.6 mg, 0.1 mmol) to give a clear solution. After one week, colorless needle crystals were obtained by slow evaporation of the solvents at room temperature.

### Refinement

The H atoms were generated geometrically and refined as riding atoms, with C–H = 0.93Å for aromatic H, C–H = 0.97Å for methylene H and C–H = 0.96Å for methyl H. The *U*_iso_(H) = *xU*_eq_(C), where *x* = 1.5 for methyl H and *x* = 1.2 for all other H atoms.

### Crystal data

**Table:**

[ZnCl_2_(C_15_H_13_N_5_)_2_]	*F*(000) = 1360
*M**_r_* = 662.90	*D*_x_ = 1.527 Mg m^−^^3^
Monoclinic, *C*2/*c*	Mo *K*α radiation, λ = 0.71073 Å
Hall symbol: -C 2yc	Cell parameters from 3926 reflections
*a* = 15.721 (4) Å	θ = 2.1–27.9°
*b* = 12.617 (4) Å	µ = 1.08 mm^−^^1^
*c* = 14.728 (3) Å	*T* = 295 K
β = 99.13 (3)°	Needle, colourless
*V* = 2884.3 (13) Å^3^	0.22 × 0.20 × 0.20 mm
*Z* = 4	

### Data collection

**Table:**

Rigaku Saturn CCD diffractometer	3423 independent reflections
Radiation source: fine-focus sealed tube	2977 reflections with *I* > 2σ(*I*)
Graphite monochromator	*R*_int_ = 0.041
Detector resolution: 28.5714 pixels mm^-1^	θ_max_ = 27.9°, θ_min_ = 2.4°
ω scans	*h* = −20→20
Absorption correction: multi-scan (*CrystalClear*; Rigaku/MSC, 2006)	*k* = −16→16
*T*_min_ = 0.797, *T*_max_ = 0.813	*l* = −19→19
17560 measured reflections	

### Refinement

**Table:**

Refinement on *F*^2^	Primary atom site location: structure-invariant direct methods
Least-squares matrix: full	Secondary atom site location: difference Fourier map
*R*[*F*^2^ > 2σ(*F*^2^)] = 0.050	Hydrogen site location: inferred from neighbouring sites
*wR*(*F*^2^) = 0.137	H-atom parameters constrained
*S* = 1.07	*w* = 1/[σ^2^(*F*_o_^2^) + (0.0749*P*)^2^ + 0.7625*P*] where *P* = (*F*_o_^2^ + 2*F*_c_^2^)/3
3423 reflections	(Δ/σ)_max_ < 0.001
196 parameters	Δρ_max_ = 0.25 e Å^−^^3^
0 restraints	Δρ_min_ = −0.30 e Å^−^^3^

### Special details

**Table:**

Geometry. All s.u.'s (except the s.u. in the dihedral angle between two l.s. planes) are estimated using the full covariance matrix. The cell s.u.'s are taken into account individually in the estimation of s.u.'s in distances, angles and torsion angles; correlations between s.u.'s in cell parameters are only used when they are defined by crystal symmetry. An approximate (isotropic) treatment of cell s.u.'s is used for estimating s.u.'s involving l.s. planes.
Refinement. Refinement of *F*^2^ against ALL reflections. The weighted *R*-factor *wR* and goodness of fit *S* are based on *F*^2^, conventional *R*-factors *R* are based on *F*, with *F* set to zero for negative *F*^2^. The threshold expression of *F*^2^ > σ(*F*^2^) is used only for calculating *R*-factors(gt) *etc*. and is not relevant to the choice of reflections for refinement. *R*-factors based on *F*^2^ are statistically about twice as large as those based on *F*, and *R*-factors based on ALL data will be even larger.

### Fractional atomic coordinates and isotropic or equivalent isotropic displacement parameters (Å^2^)

**Table:**

	*x*	*y*	*z*	*U*_iso_*/*U*_eq_	
Zn1	0.0000	0.58999 (3)	0.2500	0.04555 (17)	
N1	0.02430 (13)	0.48160 (16)	0.15242 (14)	0.0412 (5)	
N2	0.08873 (13)	0.35668 (17)	0.08201 (15)	0.0421 (5)	
N3	0.14451 (15)	0.18031 (19)	0.08324 (15)	0.0489 (5)	
N4	0.14628 (19)	0.1031 (2)	0.01911 (17)	0.0623 (7)	
N5	0.13466 (19)	0.0122 (2)	0.05545 (18)	0.0653 (7)	
C1	−0.03854 (16)	0.42678 (18)	0.09166 (16)	0.0379 (5)	
C2	−0.12745 (17)	0.4407 (2)	0.07150 (18)	0.0472 (6)	
H2	−0.1550	0.4934	0.1000	0.057*	
C3	−0.17295 (18)	0.3735 (3)	0.0079 (2)	0.0525 (7)	
H3	−0.2325	0.3805	−0.0062	0.063*	
C4	−0.13169 (19)	0.2953 (2)	−0.0360 (2)	0.0567 (7)	
H4	−0.1646	0.2515	−0.0787	0.068*	
C5	−0.04384 (19)	0.2807 (2)	−0.01797 (18)	0.0493 (6)	
H5	−0.0163	0.2288	−0.0475	0.059*	
C6	0.00111 (16)	0.34824 (19)	0.04711 (16)	0.0387 (5)	
C7	0.09879 (16)	0.4373 (2)	0.14406 (17)	0.0417 (6)	
C8	0.18438 (18)	0.4695 (3)	0.1936 (2)	0.0620 (8)	
H8A	0.1787	0.5340	0.2268	0.093*	
H8B	0.2230	0.4808	0.1502	0.093*	
H8C	0.2068	0.4147	0.2360	0.093*	
C9	0.15636 (18)	0.2894 (2)	0.0572 (2)	0.0509 (7)	
H9A	0.1560	0.2934	−0.0086	0.061*	
H9B	0.2119	0.3146	0.0878	0.061*	
C10	0.13101 (16)	0.1366 (2)	0.16454 (17)	0.0454 (6)	
C11	0.1241 (2)	0.1776 (3)	0.25161 (19)	0.0562 (7)	
H11	0.1292	0.2497	0.2644	0.067*	
C12	0.1096 (2)	0.1053 (3)	0.3164 (2)	0.0637 (9)	
H12	0.1046	0.1293	0.3750	0.076*	
C13	0.10205 (19)	−0.0044 (3)	0.2980 (2)	0.0668 (9)	
H13	0.0923	−0.0508	0.3444	0.080*	
C14	0.1089 (2)	−0.0433 (3)	0.2129 (2)	0.0633 (8)	
H14	0.1034	−0.1154	0.2002	0.076*	
C15	0.12426 (18)	0.0289 (2)	0.14573 (19)	0.0512 (7)	
Cl1	−0.11365 (6)	0.69095 (6)	0.19298 (5)	0.0643 (3)	

### Atomic displacement parameters (Å^2^)

**Table:**

	*U*^11^	*U*^22^	*U*^33^	*U*^12^	*U*^13^	*U*^23^
Zn1	0.0627 (3)	0.0303 (2)	0.0417 (3)	0.000	0.0021 (2)	0.000
N1	0.0454 (11)	0.0342 (10)	0.0424 (11)	−0.0021 (9)	0.0020 (9)	−0.0038 (9)
N2	0.0410 (11)	0.0413 (11)	0.0447 (12)	0.0035 (9)	0.0089 (9)	−0.0003 (9)
N3	0.0578 (14)	0.0496 (13)	0.0404 (12)	0.0149 (11)	0.0118 (10)	−0.0014 (10)
N4	0.0825 (19)	0.0610 (16)	0.0442 (13)	0.0202 (14)	0.0125 (13)	−0.0077 (11)
N5	0.0884 (19)	0.0519 (15)	0.0553 (15)	0.0168 (14)	0.0102 (13)	−0.0051 (12)
C1	0.0421 (13)	0.0353 (11)	0.0356 (12)	−0.0016 (10)	0.0043 (10)	0.0002 (9)
C2	0.0445 (14)	0.0487 (14)	0.0481 (15)	0.0018 (11)	0.0065 (11)	−0.0033 (12)
C3	0.0403 (14)	0.0606 (17)	0.0552 (16)	−0.0069 (13)	0.0038 (12)	−0.0026 (14)
C4	0.0582 (18)	0.0581 (17)	0.0518 (16)	−0.0180 (14)	0.0024 (13)	−0.0129 (13)
C5	0.0578 (17)	0.0447 (14)	0.0461 (14)	−0.0049 (12)	0.0108 (12)	−0.0100 (11)
C6	0.0444 (13)	0.0355 (12)	0.0367 (12)	−0.0022 (10)	0.0078 (10)	0.0021 (9)
C7	0.0410 (13)	0.0404 (13)	0.0427 (13)	−0.0014 (10)	0.0039 (10)	0.0008 (11)
C8	0.0474 (16)	0.070 (2)	0.0648 (19)	−0.0058 (14)	−0.0036 (14)	−0.0091 (16)
C9	0.0498 (15)	0.0562 (16)	0.0497 (15)	0.0084 (13)	0.0172 (12)	0.0017 (13)
C10	0.0432 (13)	0.0531 (15)	0.0397 (13)	0.0127 (12)	0.0062 (11)	0.0013 (11)
C11	0.0612 (18)	0.0644 (18)	0.0431 (15)	0.0068 (14)	0.0089 (13)	−0.0028 (13)
C12	0.0542 (17)	0.098 (3)	0.0389 (15)	0.0078 (17)	0.0077 (13)	0.0052 (16)
C13	0.0517 (17)	0.084 (2)	0.063 (2)	0.0022 (16)	0.0031 (14)	0.0250 (18)
C14	0.0589 (18)	0.0569 (18)	0.071 (2)	0.0042 (15)	0.0001 (15)	0.0115 (16)
C15	0.0485 (15)	0.0522 (16)	0.0514 (15)	0.0120 (12)	0.0033 (12)	0.0016 (13)
Cl1	0.0925 (6)	0.0456 (4)	0.0524 (4)	0.0254 (4)	0.0040 (4)	0.0033 (3)

### Geometric parameters (Å, º)

**Table:**

Zn1—N1^i^	2.063 (2)	C4—C5	1.377 (4)
Zn1—N1	2.063 (2)	C4—H4	0.9300
Zn1—Cl1^i^	2.2458 (9)	C5—C6	1.389 (3)
Zn1—Cl1	2.2458 (9)	C5—H5	0.9300
N1—C7	1.321 (3)	C7—C8	1.482 (4)
N1—C1	1.405 (3)	C8—H8A	0.9600
N2—C7	1.359 (3)	C8—H8B	0.9600
N2—C6	1.396 (3)	C8—H8C	0.9600
N2—C9	1.451 (3)	C9—H9A	0.9700
N3—N4	1.360 (3)	C9—H9B	0.9700
N3—C10	1.365 (3)	C10—C15	1.388 (4)
N3—C9	1.449 (3)	C10—C11	1.403 (4)
N4—N5	1.290 (4)	C11—C12	1.366 (4)
N5—C15	1.381 (4)	C11—H11	0.9300
C1—C6	1.390 (3)	C12—C13	1.412 (5)
C1—C2	1.393 (4)	C12—H12	0.9300
C2—C3	1.376 (4)	C13—C14	1.366 (5)
C2—H2	0.9300	C13—H13	0.9300
C3—C4	1.395 (4)	C14—C15	1.395 (4)
C3—H3	0.9300	C14—H14	0.9300
			
N1^i^—Zn1—N1	96.95 (12)	C1—C6—N2	105.2 (2)
N1^i^—Zn1—Cl1^i^	109.86 (6)	N1—C7—N2	111.7 (2)
N1—Zn1—Cl1^i^	114.34 (6)	N1—C7—C8	125.9 (2)
N1^i^—Zn1—Cl1	114.34 (6)	N2—C7—C8	122.4 (2)
N1—Zn1—Cl1	109.86 (6)	C7—C8—H8A	109.5
Cl1^i^—Zn1—Cl1	110.89 (5)	C7—C8—H8B	109.5
C7—N1—C1	106.0 (2)	H8A—C8—H8B	109.5
C7—N1—Zn1	127.62 (17)	C7—C8—H8C	109.5
C1—N1—Zn1	125.50 (16)	H8A—C8—H8C	109.5
C7—N2—C6	107.9 (2)	H8B—C8—H8C	109.5
C7—N2—C9	126.5 (2)	N3—C9—N2	111.0 (2)
C6—N2—C9	125.6 (2)	N3—C9—H9A	109.4
N4—N3—C10	110.1 (2)	N2—C9—H9A	109.4
N4—N3—C9	118.6 (2)	N3—C9—H9B	109.4
C10—N3—C9	131.3 (2)	N2—C9—H9B	109.4
N5—N4—N3	109.2 (2)	H9A—C9—H9B	108.0
N4—N5—C15	108.1 (2)	N3—C10—C15	103.8 (2)
C6—C1—C2	119.9 (2)	N3—C10—C11	134.3 (3)
C6—C1—N1	109.2 (2)	C15—C10—C11	121.9 (3)
C2—C1—N1	130.8 (2)	C12—C11—C10	116.0 (3)
C3—C2—C1	117.6 (3)	C12—C11—H11	122.0
C3—C2—H2	121.2	C10—C11—H11	122.0
C1—C2—H2	121.2	C11—C12—C13	122.7 (3)
C2—C3—C4	121.4 (3)	C11—C12—H12	118.7
C2—C3—H3	119.3	C13—C12—H12	118.7
C4—C3—H3	119.3	C14—C13—C12	120.7 (3)
C5—C4—C3	122.1 (3)	C14—C13—H13	119.6
C5—C4—H4	119.0	C12—C13—H13	119.6
C3—C4—H4	119.0	C13—C14—C15	117.7 (3)
C4—C5—C6	115.8 (3)	C13—C14—H14	121.2
C4—C5—H5	122.1	C15—C14—H14	121.2
C6—C5—H5	122.1	N5—C15—C10	108.9 (2)
C5—C6—C1	123.1 (2)	N5—C15—C14	130.1 (3)
C5—C6—N2	131.7 (2)	C10—C15—C14	121.0 (3)
			
N1^i^—Zn1—N1—C7	−88.2 (2)	Zn1—N1—C7—N2	169.67 (16)
Cl1^i^—Zn1—N1—C7	27.3 (2)	C1—N1—C7—C8	178.9 (3)
Cl1—Zn1—N1—C7	152.8 (2)	Zn1—N1—C7—C8	−11.2 (4)
N1^i^—Zn1—N1—C1	79.87 (19)	C6—N2—C7—N1	0.2 (3)
Cl1^i^—Zn1—N1—C1	−164.61 (17)	C9—N2—C7—N1	−178.6 (2)
Cl1—Zn1—N1—C1	−39.2 (2)	C6—N2—C7—C8	−179.0 (3)
C10—N3—N4—N5	−0.1 (3)	C9—N2—C7—C8	2.2 (4)
C9—N3—N4—N5	180.0 (3)	N4—N3—C9—N2	129.2 (3)
N3—N4—N5—C15	0.4 (4)	C10—N3—C9—N2	−50.8 (4)
C7—N1—C1—C6	0.1 (3)	C7—N2—C9—N3	115.7 (3)
Zn1—N1—C1—C6	−170.04 (15)	C6—N2—C9—N3	−62.9 (3)
C7—N1—C1—C2	−179.1 (3)	N4—N3—C10—C15	−0.3 (3)
Zn1—N1—C1—C2	10.7 (4)	C9—N3—C10—C15	179.7 (3)
C6—C1—C2—C3	0.9 (4)	N4—N3—C10—C11	179.5 (3)
N1—C1—C2—C3	−179.9 (3)	C9—N3—C10—C11	−0.6 (5)
C1—C2—C3—C4	−0.9 (4)	N3—C10—C11—C12	179.7 (3)
C2—C3—C4—C5	0.2 (5)	C15—C10—C11—C12	−0.6 (4)
C3—C4—C5—C6	0.5 (4)	C10—C11—C12—C13	0.1 (4)
C4—C5—C6—C1	−0.5 (4)	C11—C12—C13—C14	−0.2 (5)
C4—C5—C6—N2	−179.9 (3)	C12—C13—C14—C15	0.6 (4)
C2—C1—C6—C5	−0.2 (4)	N4—N5—C15—C10	−0.6 (3)
N1—C1—C6—C5	−179.5 (2)	N4—N5—C15—C14	179.0 (3)
C2—C1—C6—N2	179.4 (2)	N3—C10—C15—N5	0.6 (3)
N1—C1—C6—N2	0.0 (3)	C11—C10—C15—N5	−179.3 (3)
C7—N2—C6—C5	179.3 (3)	N3—C10—C15—C14	−179.1 (3)
C9—N2—C6—C5	−1.8 (4)	C11—C10—C15—C14	1.1 (4)
C7—N2—C6—C1	−0.1 (3)	C13—C14—C15—N5	179.4 (3)
C9—N2—C6—C1	178.7 (2)	C13—C14—C15—C10	−1.0 (4)
C1—N1—C7—N2	−0.2 (3)		

Symmetry code: (i) −*x*, *y*, −*z*+1/2.
